# Differential survival benefit of curative versus non-curative intent treatment in a real-world cohort with early and intermediate-stage hepatocellular carcinoma

**DOI:** 10.1097/HC9.0000000000000891

**Published:** 2026-01-29

**Authors:** David Goldberg, Binu John, David Kaplan, Cindy Delgado, Neelima Gaddipati, Sirisha Gaddipati, Catherine Blandon

**Affiliations:** 1 Division of Digestive Health and Liver Diseases, Department of Medicine, University of Miami Miller School of Medicine, Miami, Florida, USA; 2 Department of Public Health Sciences, University of Miami Miller School of Medicine, Miami, Florida, USA; 3 Bruce W. Carter Department of Veterans Affairs Medical Center, Miami, Florida, USA; 4 Division of Gastroenterology, Department of Medicine, University of Pennsylvania Perelman School of Medicine, Philadelphia, Pennsylvania, USA; 5 Corporal Michael J. Crescenz Department of Veterans Affairs Medical Center, Philadelphia, Pennsylvania, USA; 6 Department of Medicine, Jackson Memorial, Miami, Florida, USA

**Keywords:** benefit, curative, survival

## Abstract

**Background:**

Treatment algorithms for hepatocellular carcinoma (HCC) account for tumor burden/stage and severity of liver disease. There are conflicting data on the differential benefit of curative versus non-curative treatments based on BCLC stage. Therefore, we sought to leverage a nationally representative sample of patients in the Veterans Health Administration (VHA) to address this question.

**Methods:**

We performed a retrospective cohort study of patients with cirrhosis and HCC in the VHA. We conducted a landmark analysis and fit survival models, censored at 3 years, to evaluate the association of treatment type based on the highest level (non-curative vs. curative vs. combination), stratified by BCLC stage, for patients with early-stage to intermediate-stage HCC.

**Results:**

We evaluated 1191 patients with confirmed HCC (535 received only non-curative treatment, 227 only curative, and 429 received a combination of curative and non-curative). Among BCLC-0 patients, patients who received curative-intent therapy had significantly better survival at all time points compared with patients receiving non-curative treatment only (HR ranging from 0.53 at 6 months to 0.77 at 3 years). In contrast, for patients with BCLC-A stage disease, receiving either curative treatment alone or combination therapy was associated with significantly better survival compared with non-curative treatment (HR ranged from 0.41 to 0.76 over the study period). However, for BCLC-B stage disease, only combination therapy had significantly better survival (HR 0.44 at 6 months to 0.65 at 3 years).

**Conclusions:**

Our real-world data demonstrate that among patients with early-stage to intermediate-stage HCC, curative-intent treatment is associated with the best survival for patients with BCLC-0 and BCLC-A stage disease, while combination therapy yields the best outcomes in patients with BCLC-B stage disease.

## INTRODUCTION

Treatment algorithms for patients with hepatocellular carcinoma (HCC) account for both tumor burden/stage and severity of underlying liver disease. There are several published staging and treatment strategies for patients with HCC, with the most commonly cited being the Barcelona Clinic Liver Cancer (BCLC) criteria.^1^ For patients with very early stage (BCLC-0) or early stage (BCLC-A) disease, first-line treatment recommendations focus on potentially curative treatment (ie, ablation, resection, or transplant), with non-curative loco-regional therapies recommended as the first-line for intermediate-stage disease (BCLC-B). Non-curative treatments [eg, transarterial chemoembolization (TACE) or transarterial radioembolization (TARE)] are considered second-line for very early-stage or early-stage disease when one of the curative options is not feasible or unsuccessful. However, in clinical practice, patients often do not receive treatment in line with these recommendations for a number of reasons, including the availability of local expertise and access to multi-disciplinary liver tumor boards.^2^^–^^4^

Despite an extensive body of literature on the outcomes associated with loco-regional and surgical treatments for HCC, there are few randomized controlled trials (RCTs) comparing one treatment to another. Studies comparing the survival benefit of different treatment strategies for HCC have largely relied on observational studies with inherent selection biases.^3^^–^^11^ Even among the small subset of RCTs that compared 2 treatment options, none have compared all of the different treatment options in a cohort with a similar HCC stage.^10^ Because of conflicting data in the literature, treatment for HCC is largely categorized into 3 groups: (a) systemic; (b) non-curative loco-regional therapy (LRT; chemo-embolization, radio-embolization, external radiation); and (c) curative (liver transplantation, resection, ablation).^4^

Due to study heterogeneity and selection bias in retrospective studies, in a field with constantly evolving treatment modalities, there are conflicting data on the differential survival benefit of curative versus non-curative treatments based on BCLC stage. Therefore, we sought to leverage a nationally representative sample of patients with cirrhosis and HCC in the Veterans Health Administration (VHA) to evaluate the benefit of different HCC treatment modalities, accounting for tumor stage, severity of liver disease, and key medical comorbidities.

## METHODS

### Patient cohort

The data were obtained from the VHA for a series of studies related to risk prediction among patients with cirrhosis and HCC.^12^^,^^13^ In short, the cohort included VHA patients with an incident diagnosis of HCC from January 24, 2014, to June 9, 2021. The cohort was restricted to patients aged 18–75 years with cirrhosis based on validated International Classification of Diseases (ICD) codes,^3^^,^^14^^–^^18^ with a confirmed diagnosis of HCC. The diagnosis of HCC was based on an initial screening using ICD codes, followed by manual chart abstraction that confirmed HCC based on a liver biopsy or imaging report of a Liver Imaging Reporting and Data System (LI-RADS) 5 lesion from a radiology report and/or HCC Tumor Board.^19^ The cohort was limited to patients presenting for the first time with early-stage to intermediate-stage HCC limited to the liver without macrovascular invasion (BCLC-0, BCLC-A, and BCLC-B), and without major life-limiting medical comorbidities (eg, heart failure with reduced ejection fraction), who also had an Eastern Cooperative Oncology Group functional status ≤2.^12^^,^^13^^,^^20^^–^^24^ Because our objective was to assess the differential survival benefit of curative versus non-curative treatment based on HCC stage, we excluded patients who received no treatment.

### Study outcome

The outcome was overall survival 3 years after a confirmed diagnosis of HCC. The index date was determined as the earliest date of curative or non-curative treatment for HCC after a confirmed diagnosis of HCC, either by biopsy or LI-RADS-5 lesion. Patient mortality was ascertained using the VA Informatics and Computing Infrastructure (VINCI) Vital Status Master File that contained dates of death as recorded in all the main federal mortality databases. Patients were censored at the time of liver transplant and 30 days after the latter of their last follow-up or pharmacy visit.

### Study covariates

The main covariate of interest was HCC treatment, categorized as curative, non-curative, or both.^1^^,^^4^ HCC treatment was identified based on Current Procedural Technology (CPT) coding and medication coding (Supplemental Table S1, http://links.lww.com/HC9/C240). The classification of curative (transplant, resection, ablation) versus non-curative (chemo-embolization, radio-embolization, external beam radiation, systemic chemotherapy) is consistent with prior studies.^1^^,^^4^ Patients were categorized based on the totality of the treatment they received: curative only, non-curative only, or curative and non-curative.

We included a series of covariates that could be associated with the decision to treat (or not) a patient with HCC, as well as factors associated with survival among patients with cirrhosis and HCC. The covariates of interest were based at the time of HCC diagnosis: demographics (eg, age), complications of portal hypertension (eg, ascites, hepatic encephalopathy), labs [eg, international normalized ratio (INR), total bilirubin, estimated glomerular filtration rate (eGFR)], liver disease etiology, diabetes, ongoing alcohol use [Alcohol-Use Disorders Identification Test—Consumption (AUDIT-C)], medical comorbidity index using the Cirrhosis Comorbidity score (CirCom), HCC-specific risk score [eg, MIami Liver Cancer Estimator of Survival (MILES)], Child–Turcotte–Pugh (CTP) score, and specific HCC tumor data [eg, alpha-fetoprotein (AFP), total tumor diameter].^12^^,^^13^^,^^20^^,^^21^^,^^23^^–^^27^ Covariates such as etiology of liver disease and CTP score were collected by a combination of laboratory, diagnosis, and procedure codes using validated methods.^27^^–^^29^

### Statistical analysis

Patient characteristics were summarized as median or count and compared between those treated and untreated using the Kruskal–Wallis test or Chi-squared test, as appropriate. If any count was <5, the Fisher exact test was applied, using a simulated *p*-value based on 5000 iterations. This was repeated for each BCLC stage. Before model fitting, collinearity among candidate covariates was assessed using Pearson correlation coefficients (*r*). If *r*>0.7, both variables were not used in the same model. Multinomial logistic regression models were then fitted using demographics and clinical variables selected a priori and univariable-significant variables from Tables 1 and 2 to estimate the probability of assignment to each treatment tier. After model fitting, variance inflation factors (VIFs<10) and condition indices (<30) were evaluated to confirm model stability and absence of multicollinearity. Any variable indicative of multicollinearity was removed, and the model was refitted. The predicted probabilities from these models were subsequently used to calculate inverse probability of treatment weights (IPTWs) for weighted survival analyses. A density graph and histogram displayed the propensity score for each treatment tier. Moreover, a Love plot assessed covariate balance between treatment groups with Absolute Standardized Mean Differences and Kolmogorov–Smirnov Statistics.

**TABLE 1:** Clinical and demographic characteristics of patients with HCC

Variable^a^	Non-curative (N=535)	Curative (N=227)	Both (N=429)	*p*
Age (y)	66.0 [62.0, 69.0]	66.0 [63.0, 69.0]	66.0 [62.0, 69.0]	0.53
Male	531 (99.3)	224 (98.7)	423 (98.6)	0.59
Race/ethnicity				0.18
White	292 (54.6)	129 (56.8)	268 (62.5)	
Asian or Pacific Islander	15 (2.8)	6 (2.6)	5 (1.2)	
Black	146 (27.3)	57 (25.1)	94 (21.9)	
Hispanic	35 (6.5)	14 (6.2)	34 (7.9)	
Other/unknown	47 (8.8)	21 (9.3)	28 (6.5)	
AFP (ng/mL)	11.5 [5.2, 71.0]	5.5 [3.5, 11.0]	6.9 [4.0, 19.4]	<0.001
INR	1.1 [1.0, 1.3]	1.1 [1.0, 1.2]	1.1 [1.0, 1.2]	0.001
Sodium (mmol/L)	138 [136, 140]	139 [137, 140]	138 [136, 140]	0.87
Albumin (g/dL)	3.7 [3.2, 4.0]	3.7 [3.3, 4.1]	3.8 [3.4, 4.1]	<0.001
Platelet (1000/µL)	137 [95, 189]	156 [105, 210]	143 [92, 197]	0.02
Total bilirubin (mg/dL)	0.8 [0.6, 1.3]	0.7 [0.5, 1.2]	0.7 [0.6, 1.2]	0.004
eGFR (mL/min/1.73 m^2^)	91.9 [71.6, 99.5]	85.1 [72.7, 97.9]	91.1 [70.5, 98.7]	0.48
Etiology				0.66
EtOH	76 (14.2)	28 (12.3)	59 (13.8)	
EtOH+HCV	200 (37.4)	74 (32.6)	161 (37.5)	
HCV	173 (32.3)	87 (38.3)	136 (31.7)	
NAFLD-NASH	66 (12.3)	32 (14.1)	62 (14.5)	
Other	20 (3.7)	6 (2.6)	11 (2.6)	
Ascites	26 (4.9)	8 (3.5)	11 (2.6)	0.17
Hepatic encephalopathy	15 (2.8)	8 (3.5)	7 (1.6)	0.29
SBP	5 (0.9)	1 (0.4)	0 (0)	0.01
Varices	26 (4.9)	14 (6.2)	26 (6.1)	0.65
Diabetes	272 (50.8)	130 (57.3)	261 (60.8)	0.007

^a^ Median (IQR) for continuous variables or N (%) for categorical variables.

Abbreviations: AFP, alpha-fetoprotein; eGFR, estimated glomerular filtration rate; EtOH, alcohol (ethanol); HCC, hepatocellular carcinoma; HCV, hepatitis C virus; INR, international normalized ratio; NASH, nonalcoholic steatohepatitis; NAFLD, nonalcoholic fatty liver disease; SBP, spontaneous bacterial peritonitis.

**TABLE 2:** HCC and comorbidities of patients with HCC

Variable^a^	Non-curative (N=535)	Curative (N=227)	Both (N=429)	*p*
Number of tumors	1.0 [1.0, 2.0]	1.0 [1.0, 1.0]	1.0 [1.0, 1.0]	<0.001
Total tumor size (cm)	3.8 [2.5, 6.1]	2.2 [1.7, 3.1]	2.7 [2.0, 4.0]	<0.001
Largest tumor (cm)	3.1 [2.3, 4.7]	2.2 [1.7, 2.9]	2.5 [1.9, 3.4]	<0.001
BCLC				<0.001
BCLC-0	66 (12.3)	72 (31.7)	100 (23.3)	
BCLC-A	295 (55.1)	140 (61.7)	277 (64.6)	
BCLC-B	174 (32.5)	15 (6.6)	52 (12.1)	
Cirrhosis Comorbidity (CirCom)^b^				0.003
0	31 (5.8)	12 (5.3)	19 (4.4)	
1+0	131 (24.5)	55 (24.2)	68 (15.9)	
1+1	123 (23.0)	54 (23.8)	103 (24.0)	
3+0	17 (3.2)	8 (3.5)	18 (4.2)	
3+1	203 (37.9)	96 (42.3)	211 (49.2)	
5+0	11 (2.1)	0 (0)	4 (0.9)	
5+1	19 (3.6)	2 (0.9)	6 (1.4)	
MILES	6.8 [6.3, 7.2]	7.2 [6.9, 7.6]	7.2 [6.8, 7.6]	<0.001
ALBI grade				0.002
Grade 1	173 (32.3)	95 (41.9)	178 (41.5)	
Grade 2	321 (60.0)	113 (49.8)	234 (54.5)	
Grade 3	41 (7.7)	19 (8.4)	17 (4.0)	
Transplant	0 (0)	9 (4.0)	50 (11.7)	
Hepatectomy	0 (0)	83 (36.6)	55 (12.8)	
Ablation	0 (0)	150 (66.1)	359 (83.7)	
HCC Oral/IV	195 (36.4)	0 (0%)	111 (25.9)	
Radiation	67 (12.5)	0 (0%)	58 (13.5)	
Embolization	437 (81.7)	0 (0%)	384 (89.5)	
Time to treatment	75.0 [39.5, 167.5]	67.0 [45.0, 116.0]	289.0 [111.0, 650.0]	<0.001
Deaths within 3 years after HCC therapy	362 (67.7)	83 (36.6)	191 (44.5)	<0.001

^a^ Median (IQR) for continuous variables or N (%) for categorical variables.

^b^ The CirCom score is a comorbidity index developed by Jepsen et al.^25^

Abbreviations: ALBI, albumin–bilirubin; BCLC, Barcelona Clinic Liver Cancer; HCC, hepatocellular carcinoma; IV, intravenous; MILES, MIami Liver Cancer Estimator of Survival.

The analyses used a landmark analysis framework, whereby we estimated time-to-event probabilities for each group conditional on their membership (ie, treatment category) at a landmark time. For patients who received only curative or non-curative treatment, follow-up (ie, landmark time) began on the date of their first treatment; for patients who received combination therapy, follow-up began on the date of the second treatment. This approach estimated follow-up time under a landmark analysis framework, removing immortal time bias because follow-up began on entry into a specific group.^30^ In addition, we conducted 2 separate analyses: one excluding patients who received only chemotherapy, and another excluding those who underwent curative treatment followed by non-curative therapy to further explore treatment impacts. We excluded patients who only received chemotherapy to limit the non-curative cohort to those who received some form of loco-regional therapy, who may have inherent differences from those who received chemotherapy. We excluded the small subset who received curative treatment first to determine whether the ordering of combination treatment impacted outcomes.

The optimal survival distribution was first identified by comparing alternative parametric forms (exponential, Gaussian, logistic, log-logistic, log-normal, and Weibull) and selecting the distribution with the lowest Akaike Information Criterion (AIC) and Bayesian Information Criterion (BIC). This distribution was then applied in a flexible parametric survival model to estimate the association between HCC treatment and survival for each BCLC stage. Schoenfeld residuals test and graphs examined the proportional hazards assumption.

After fitting models only for baseline covariates, without accounting for factors associated with treatment assignment, we then adjusted for MILES and contained inverse probability of treatment weighting (IPTW) to adjust for patient characteristics in the different HCC treatment tiers. The IPTW was derived from the multinomial models mentioned above and was fit with and without the CirCom score. Survival probabilities over a 3-year horizon were plotted for different HCC treatment tiers. Corresponding hazard ratios with 95% confidence intervals for HCC treatment tiers were displayed for both the baseline and adjusted models, evaluated at the 50th percentile of MILES.

All analyses were performed in R version 4.4.1 software (The R Foundation for Statistical Computing). A *p*-value <0.05 was considered significant. The study was approved by the Institutional Review Board at the University of Miami and the Bruce Carter Department of Veterans Affairs Medical Center.

## RESULTS

Among the 1325 patients with confirmed HCC identified in the study population, 1191 (89.9%) received some form of curative and/or non-curative HCC treatment and were included in the analyses. Of the 1191 patients included, 535 (40.4%) received only non-curative treatment, 227 (17.1%) received only curative treatment, and 429 (32.4%) received a combination of curative and non-curative treatment. There were no clinically meaningful differences in the clinical and demographic characteristics among the overall cohort stratified by treatment type (Tables 1 and 2). When stratified by BCLC stage, the only statistically significant clinical differences based on treatment type (albeit unclear clinical relevance) were INR and total bilirubin for BCLC-0 stage disease, AFP and platelet count for BCLC-A stage disease (Supplemental Tables S2–S4, http://links.lww.com/HC9/C241, http://links.lww.com/HC9/C242, http://links.lww.com/HC9/C243). Other than a longer time from HCC diagnosis to treatment for patients who received curative and non-curative treatment, an artifact of the landmark analysis, there were no other clinically relevant differences in the tumor characteristics based on BCLC stage and treatment type (Table 2).

### Survival based on treatment status

**FIGURE 1:**
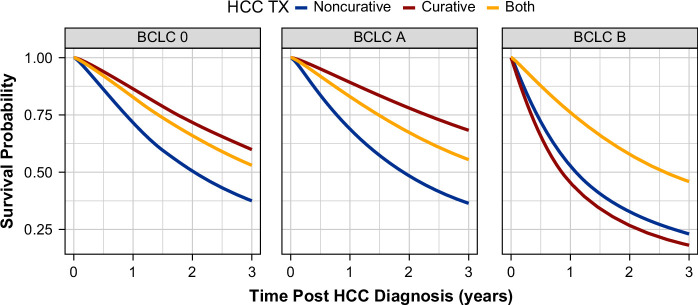
Absolute unadjusted survival based on treatment type and BCLC stage. Abbreviations: BCLC, Barcelona Clinic Liver Cancer; HCC, hepatocellular carcinoma.

The unadjusted survival based on treatment type differed based on baseline BCLC stage. For patients with BCLC-0 and BCLC-A stage disease, unadjusted survival was highest among those who received curative treatment, lowest in those who received non-curative treatment, and intermediate for patients who received both curative and non-curative treatment (Table 3 and Figure 1). However, for patients with BCLC-B stage HCC, survival was highest among patients who received combined curative and non-curative treatment, with no significant differences among those who received curative versus non-curative treatment (Table 3 and Figure 1).

**TABLE 3:** Unadjusted survival based on BCLC stage and treatment category

BCLC	Treatment	N	6 months	1 year	2 years	3 years
	Non-curative	66	0.86 (0.80, 0.92)	0.72 (0.62, 0.80)	0.50 (0.40, 0.61)	0.37 (0.27, 0.48)
0	Curative	72	0.94 (0.90, 0.97)	0.86 (0.79, 0.91)	0.72 (0.61, 0.80)	0.60 (0.48, 0.70)
	Both	100	0.92 (0.88, 0.95)	0.83 (0.76, 0.88)	0.66 (0.58, 0.74)	0.53 (0.44, 0.62)
	Non-curative	295	0.84 (0.81, 0.87)	0.69 (0.64, 0.73)	0.48 (0.43, 0.54)	0.36 (0.31, 0.42)
A	Curative	140	0.95 (0.93, 0.97)	0.89 (0.86, 0.92)	0.78 (0.72, 0.83)	0.68 (0.60, 0.75)
	Both	277	0.92 (0.90, 0.94)	0.83 (0.80, 0.86)	0.67 (0.62, 0.72)	0.56 (0.50, 0.61)
	Non-curative	174	0.72 (0.67, 0.78)	0.53 (0.46, 0.59)	0.33 (0.27, 0.39)	0.23 (0.18, 0.28)
B	Curative	15	0.66 (0.40, 0.85)	0.45 (0.23, 0.70)	0.27 (0.12, 0.51)	0.18 (0.07, 0.39)
	Both	52	0.88 (0.82, 0.93)	0.76 (0.65, 0.84)	0.58 (0.45, 0.69)	0.46 (0.34, 0.57)

Abbreviation: BCLC, Barcelona Clinic Liver Cancer.

When further stratified by BCLC stage and CTP class, a different pattern of survival differences emerged. Among BCLC-0 patients with CTP-A disease, survival was highest in patients who received curative treatment, while among CTP-B patients, it was highest among those receiving curative and non-curative treatment, and similar in those who received curative versus non-curative treatment (Table 4). A similar pattern was seen among those with BCLC-A or BCLC-B disease, when stratified by CTP class—survival was highest among patients receiving combination therapy, and not different among those receiving curative versus non-curative treatment.

**TABLE 4:** Unadjusted survival by BCLC, CTP, and treatment category

BCLC	CTP	Treatment	N	Deaths	6 months	1 year	2 years	3 years
0	A	Non-curative	46	27	0.89 (0.81, 0.94)	0.75 (0.64, 0.84)	0.55 (0.41, 0.67)	0.41 (0.29, 0.54)
		Curative	59	20	0.95 (0.92, 0.98)	0.89 (0.83, 0.93)	0.77 (0.66, 0.85)	0.66 (0.52, 0.77)
		Both	83	39	0.92 (0.88, 0.95)	0.82 (0.75, 0.88)	0.65 (0.55, 0.73)	0.52 (0.41, 0.62)
	B	Non-curative	20	16	0.82 (0.68, 0.93)	0.64 (0.46, 0.80)	0.42 (0.24, 0.60)	0.30 (0.14, 0.47)
		Curative	13	8	0.84 (0.66, 0.95)	0.68 (0.43, 0.87)	0.45 (0.21, 0.73)	0.33 (0.13, 0.61)
		Both	17	6	0.94 (0.85, 0.98)	0.87 (0.71, 0.95)	0.72 (0.46, 0.87)	0.60 (0.31, 0.80)
A	A	Non-curative	232	134	0.77 (0.71, 0.82)	0.59 (0.52, 0.66)	0.39 (0.32, 0.47)	0.29 (0.22, 0.36)
		Curative	114	29	0.72 (0.46, 0.89)	0.53 (0.26, 0.77)	0.34 (0.14, 0.60)	0.24 (0.09, 0.49)
		Both	237	96	0.90 (0.83, 0.94)	0.80 (0.70, 0.88)	0.64 (0.51, 0.75)	0.52 (0.39, 0.65)
	B	Non-curative	63	54	0.54 (0.41, 0.67)	0.27 (0.16, 0.39)	0.11 (0.05, 0.19)	0.06 (0.02, 0.12)
		Curative	26	15	0.18 (0.01, 0.86)	0.06 (0.00, 0.66)	0.02 (0.00, 0.35)	0.01 (0.00, 0.21)
		Both	40	20	0.82 (0.62, 0.93)	0.58 (0.34, 0.79)	0.31 (0.13, 0.54)	0.19 (0.07, 0.38)
B	A	Non-curative	133	94	0.77 (0.71, 0.83)	0.59 (0.52, 0.66)	0.39 (0.31, 0.46)	0.29 (0.21, 0.36)
		Curative	13	9	0.72 (0.49, 0.89)	0.53 (0.29, 0.78)	0.34 (0.15, 0.61)	0.24 (0.10, 0.49)
		Both	43	22	0.90 (0.83, 0.94)	0.80 (0.69, 0.87)	0.64 (0.51, 0.75)	0.52 (0.39, 0.65)
	B	Non-curative	41	37	0.54 (0.40, 0.67)	0.27 (0.16, 0.39)	0.11 (0.05, 0.19)	0.06 (0.02, 0.12)
		Curative	2	2	0.18 (0.01, 0.83)	0.06 (0.00, 0.61)	0.02 (0.00, 0.33)	0.01 (0.00, 0.20)
		Both	9	8	0.82 (0.62, 0.94)	0.58 (0.34, 0.80)	0.31 (0.13, 0.54)	0.19 (0.07, 0.38)

Abbreviations: BCLC, Barcelona Clinic Liver Cancer; CTP, Child–Turcotte–Pugh.

When stratified only by BCLC stage, the 6-month survival from HCC diagnosis exceeded 85% in all patients with BCLC-0 stage HCC, regardless of treatment type. By contrast, there were more marked differences (non-overlapping confidence intervals) in 6-month survival in those with BCLC-A and BCLC-B stage disease based on treatment status (Table 3 and Figure 1). These patterns of variability in survival differed when the cohort was additionally stratified based on baseline CTP score (Table 4).^27^ In comparison, among patients with BCLC-A and BCLC-B stage disease, starting at 1 year after HCC diagnosis, although the differences in survival between treatment types (none versus non-curative versus curative) were similar when stratified by CTP score, the point estimates for unadjusted survival were markedly lower for those with CTP-B (versus CTP-A) disease (Table 4).

### Multivariable models

**FIGURE 2:**
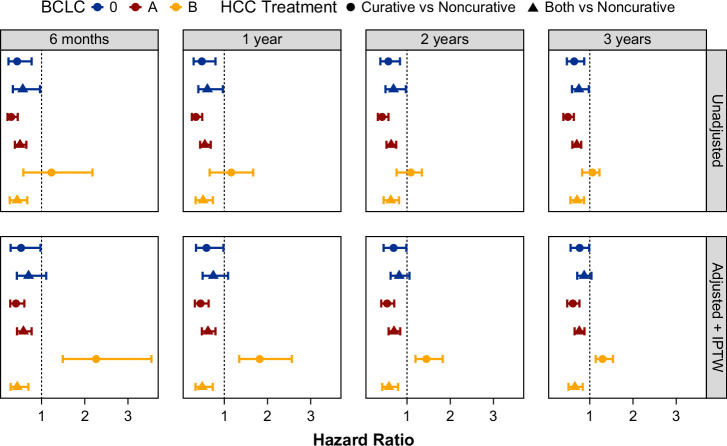
Forest plot of hazard ratios of multivariable adjusted models with and without inverse probability of treatment weighting. Abbreviations: BCLC, Barcelona Clinic Liver Cancer; HCC, hepatocellular carcinoma; IPTW, inverse probability of treatment weight.

The IPTW multinomial model adjusted for age, sex, race, diabetes, AFP, bilirubin, INR, platelets, sodium, eGFR, CirCom, ALBI grade, number of tumors, total tumor burden, BCLC, and MILES. The Absolute Standardized Mean Differences for the variables considered were all below 0.1 except for CirCom, ALBI class, and ethnicity (Supplemental Figure S1, http://links.lww.com/HC9/C244). There were small differences in the point estimates of the hazard ratios of multivariable models with baseline adjustment versus those with IPTW (Figure 2). Within each BCLC stage, there were differences in the hazard ratios as a function of treatment type and follow-up time.

Among BCLC-0 patients, there were no significant differences at any time point in the adjusted survival of those receiving combination curative + non-curative therapy, while curative treatment alone was associated with better survival at all time points compared with the reference group receiving non-curative treatment only (Supplemental Table S5, http://links.lww.com/HC9/C245, and Figure 2). In contrast, for patients with BCLC-A stage disease, receiving either curative treatment alone or combination therapy was associated with significantly better survival (lower mortality) compared with non-curative treatment, with no significant difference between curative versus combination therapy (Supplemental Table S5, http://links.lww.com/HC9/C245, and Figure 2). However, for BCLC-B stage disease, receiving curative treatment only had a numerically higher risk of mortality compared with non-curative only treatment, while combination therapy had significantly better survival (lower mortality) compared with patients who received only non-curative treatment (Supplemental Table S5, http://links.lww.com/HC9/C245, and Figure 2).

### Sensitivity analysis

When patients who only received systemic chemotherapy (oral and/or intravenous) as their HCC treatment were excluded from the non-curative treatment group, the point estimates for the hazard ratios remained essentially unchanged (Supplemental Tables S6 http://links.lww.com/HC9/C258, Supplemental Table S7 http://links.lww.com/HC9/C259). When the combination treatment group was limited to those who received non-curative treatment followed by curative treatment, excluding those whose first treatment was curative, the point estimates for the hazard ratios decreased (ie, better survival; Supplemental Table S5, http://links.lww.com/HC9/C245).

## DISCUSSION

In this analysis of more than 1100 patients with cirrhosis and HCC confined to the liver, we highlight the differences in survival based on treatment type (ie, curative vs. non-curative) and BCLC stage. After performing IPTW to account for the non-randomized nature of the study, we demonstrated that 3-year survival was only superior for patients with BCLC-0 stage disease if they received curative-intent treatment, while curative-intent or combination therapy was superior to non-curative for BCLC-A stage disease, and combination curative plus non-curative treatment was superior to either treatment alone for BCLC-B stage disease. This study provides important data to inform treatment decision-making for patients with cirrhosis and HCC, while also raising the question of whether the BCLC treatment algorithm should be revisited, especially for BCLC-0 and BCLC-B stage disease.

There have been a number of studies exploring the benefits of different treatment options among patients with HCC, accounting for tumor burden and severity of underlying liver disease. A major distinction in treatment decision-making is whether it is considered as having a “curative intent,” which is a function of the burden of HCC as well as the treatment that is administered. For example, a patient with a single 1.5 cm lesion who received TACE could be considered as having curative stage disease, but received non-curative treatment. Conversely, a patient with bilobar multifocal HCC who undergoes resection would receive a treatment type categorized as “curative,” but it would not be treatment with a curative intent, given the burden of disease and type of treatment. Importantly, nearly all studies comparing outcomes across different HCC treatment modalities have been non-randomized and retrospective in nature, and among prospective studies, none have randomized patients to curative versus non-curative treatments. In addition, many of these studies did not stratify by BCLC stage, and some did not account for baseline differences in characteristics that would impact treatment decision-making by utilizing IPTW. As a result, HCC treatment algorithms have largely relied on retrospective studies with inherent limitations.

Our study provides important data to further inform the discussion of the differential benefit of curative versus non-curative treatment among patients with HCC. First, in the setting of retrospective cohort studies evaluating different treatment types, a major limiting factor is the confounding by indication, whereby clinical factors influence what treatment a patient receives, which may then impact the outcome (ie, survival). We sought to account for this using IPTW, a statistical method that was not used in several of the prior studies of HCC treatment, allowing us to better account for disease severity influencing both the exposure (treatment type) and the outcome (survival). Second, by leveraging data from the VHA, we provide more real-world data from a variety of clinical sites (ie, community versus tertiary care academic), in a closed healthcare system where treatment considerations are not constrained by the payer in the same manner as the private sector. Third, we adjusted for survival by not only accounting for traditional risk factors such as age and laboratory values, but also leveraging a new risk score developed by our group that specifically focuses on overall survival among patients with HCC.^12^ Lastly, by including a modern cohort of data, we were able to include a broader array of treatment types that had not been considered in some prior studies (eg, TARE was not included in several prior studies using large databases^5^^–^^8^).

The BCLC treatment algorithm recommends curative treatment as first-line for BCLC-0 and BCLC-A disease, and either transplant, TACE, or systemic treatment for BCLC-B stage disease based on tumor burden. Our data would suggest that the BCLC algorithm should be re-evaluated, especially for those with BCLC-0 or BCLC-B stage disease. The benefit of curative treatment as first-line for BCLC-A stage disease was confirmed in our analysis. However, there was only a benefit to curative treatment for BCLC-0 stage disease, which suggests that those who received combination therapy may have subsequently developed a second lesion. Because BCLC-0 stage disease is defined by a small tumor with preserved liver function, it is not implausible to consider that advances in loco-regional therapy, specifically TACE and TARE, may offer similar tumor control to ablative (or other curative) therapies and may be considered as alternative first-line treatments. In the setting of more advanced BCLC-B stage disease, combination therapy offered a clear survival benefit, raising the question of whether combination therapy, rather than TACE alone, should be the first-line treatment option for patients who are not transplant candidates.

Our study did have limitations. We relied on data from the VHA, which has limitations related to being primarily composed of men. However the survival data in this population mirrors that in nationally representative cohorts, thereby giving confidence of their external validity.^31^ Second, while our IPTW accounted for factors that may have influenced treatment decision-making (eg, severity of underlying liver disease, tumor burden), there are factors that we could not adjust for that may have influenced treatment decision-making, and factors we adjusted but could not meet the threshold of <0.01 for covariate balance. For example, patients may have normal synthetic function, but have portal hypertension, negative hepatic resection, and a tumor in a location precluding ablation. For such patients, given limited access to transplant in the VHA, non-curative treatment may have been the only option. While such factors could influence the type of treatment a patient received, we would not have expected it to explain our findings of superior survival of curative treatment, and if anything, would have biased our results toward the null if patients eligible for curative treatment received non-curative treatment. Third, we only focused on the receipt of treatment, and not whether the treatment was deemed successful, which could impact survival if a patient’s treatment was deemed insufficient. Fourth, we did not ascertain whether the lesions we recorded in follow-up were recurrent versus new lesions, which could have explained the results among BCLC-0 patients who received combination therapy. Fifth, patients were categorized based on the highest level of treatment, even if they received non-curative to downstage, followed by curative. We do not believe this significantly impacted our results, given the relatively similar tumor volume in patients based on treatment type. Sixth, given the very small size of certain patient subsets—such as BCLC-B patients who received curative treatment (n=15)—any conclusions regarding differences between curative and non-curative modalities within these groups should be interpreted with caution. Lastly, our study was retrospective in nature, rather than a prospective study or a randomized trial.

In conclusion, in this cohort of 1100 patients with cirrhosis and HCC in the VHA, we have demonstrated that across BCLC-0, BCLC-A, and BCLC-B stages of disease, the benefit of curative versus non-curative versus combination therapy is highly dependent on BCLC stage. These data should lead the field to reconsider current stage-specific HCC treatment algorithms to optimize outcomes for patients with HCC.

## Supplementary Material
