# Supplementary material for: Differential survival benefit of curative versus non-curative intent treatment in a real-world cohort with early and intermediate-stage hepatocellular carcinoma
Source: Hepatol Commun. 2026 Jan 29;10(2):e0891. doi: 10.1097/HC9.0000000000000891 (PMC12858220; doi:10.1097/HC9.0000000000000891)
Supplement: Supplementary file 1 [file hc9-10-e0891-s001.docx]

Supplementary Table 1: Coding criteria and classification of HCC treatments

| **HCC Treatment** | **Description (name, ICD, or CPT)** |
| --- | --- |
| Curative |  |
| Liver Transplant | OPTN/UNOS linkage |
| Hepatectomy/Resection | 47120, 47122, 47125, 47130 |
| Ablation | Microwave (more than 1 encounter with the following CTP codes: 47382, 77013, 77022, 76940, 47399),  Laparoscopic (Microwave criteria and 47370),  Open Radiofrequency Ablation (Microwave criteria and 47380) |
| Noncurative |  |
| Oral/IV | ATEZOLIZUMAB, AVASTIN, BEVACIZUMAB, CABOMETYX, CABOZANTINIB, CABOZANTINIB-S-MALATE, RAMUCIRUMAB, DURVALUMAB, IMFINZI, DURVALUMAB, IMJUDO, TREMELIMUMAB, IPILIMUMAB, KEYTRUDA, PEMBROLIZUMAB, LENVATINIB, LENVIMA, LENVATINIB MESYLATE, LYTGOBI, FUTIBATINIB, NEXAVAR, SORAFENIB TOSYLATE, NIVOLUMAB, OPDIVO, NIVOLUMAB, PEMAZYRE, PEMIGATINIB, PEMBROLIZUMAB, RAMUCIRUMAB, REGORAFENIB, SORAFENIB, STIVARGA, REGORAFENIB, TECENTRIQ, ATEZOLIZUMAB, TREMELIMUMAB-ACTL, YERVOY, IPILIMUMAB |
| Radiation | IGRT (77387, G6001, G6002, G6017),  IMRT (77385, 77386, G6015, G6016),  Intraoperative radiation (77424, 77425)  Neutron Therapy (77422, 77423),  Proton Therapy (77520, 77521, 77522, 77523, 77524, 77525),  Radiation (77401, 77403, 77404, 77406, 77408, 77409, 77411, 77413, 77414, 77402, 77407, 77412, G6003, G6004, G6005, G6006, G6007, G6008, G6009, G6010, G6011, G6012, G6013,  G6014, G6015, G6016)  SRS (77371, 77372, 77373, 77374, 77385, 77385) |
| Embolization | TACE (more than 1 encounter with the following CTP codes: 36245, 36246, 36247, 36248, 75726, 75774, 37242, 37243, 37204, 96420)  Y90 injection (TACE criteria and any of the following CTP codes: 79445, 77778, C2616, S2095, Q3001, C2699),  Y90 mapping (TACE criteria and any of the following CTP codes: 77290, 78201, 78800, 78803, 78830, 78801, 78580, A9540) |
