# Supplementary material for: Differential survival benefit of curative versus non-curative intent treatment in a real-world cohort with early and intermediate-stage hepatocellular carcinoma
Source: Hepatol Commun. 2026 Jan 29;10(2):e0891. doi: 10.1097/HC9.0000000000000891 (PMC12858220; doi:10.1097/HC9.0000000000000891)
Supplement: Supplementary file 2 [file hc9-10-e0891-s002.docx]

Supplementary Table 2. Clinical and demographic characteristics of patients with HCC, BCLC stage 0

| Variable* | Noncurative  (N=66) | Curative  (N=72) | Both  (N=100) | P-value |
| --- | --- | --- | --- | --- |
| Age (years) | 66.0 [62.0, 70.0] | 66.0 [63.0, 69.0] | 66.5 [62.0, 69.0] | 0.78 |
| Male | 66 (100) | 70 (97.2) | 97 (97.0) | 0.52 |
| Race |  |  |  | 0.30 |
| White | 34 (51.5) | 39 (54.2) | 65 (65.0) |  |
| Black | 21 (31.8) | 20 (27.8) | 23 (23.0) |  |
| Hispanic | 4 (6.1) | 3 (4.2) | 8 (8.0) |  |
| Asian or Pacific Islander | 1 (1.5) | 2 (2.8) | 1 (1.0) |  |
| Other/Unknown | 6 (9.1) | 8 (11.1) | 3 (3.0) |  |
| AFP (ng/mL) | 7.6 [4.2, 12.5] | 5.3 [3.7, 10.4] | 6.9 [4.3, 16.6] | 0.31 |
| INR | 1.2 [1.1, 1.3] | 1.1 [1.0, 1.2] | 1.1 [1.0, 1.2] | 0.06 |
| Sodium (mmol/L) | 138 [136, 141] | 138 [136, 140] | 139 [136, 140] | 0.90 |
| Albumin (g/dL) | 3.6 [3.3, 4.0] | 3.8 [3.3, 4.0] | 3.9 [3.5, 4.1] | 0.21 |
| Platelet (1000/µL) | 123 [76, 176] | 147 [105, 199] | 127 [89, 187] | 0.06 |
| Total Bilirubin (mg/dL) | 0.9 [0.7, 1.4] | 0.6 [0.5, 1.1] | 0.8 [0.6, 1.2] | 0.01 |
| eGFR (mL/min/1.73 m^2^) | 94.7 [75.7, 100.3] | 91.0 [73.8, 97.6] | 92.3 [76.5, 99.6] | 0.26 |
| Etiology |  |  |  | 0.37 |
| EtOH | 10 (15.2) | 5 (6.9) | 13 (13.0) |  |
| EtOH+HCV | 26 (39.4) | 26 (36.1) | 45 (45.0) |  |
| HCV | 17 (25.8) | 29 (40.3) | 30 (30.0) |  |
| NAFLD-NASH | 10 (15.2) | 9 (12.5) | 11 (11.0) |  |
| Other | 3 (4.5) | 3 (4.2) | 1 (1.0) |  |
| Ascites | 1 (1.5) | 1 (1.4) | 3 (3.0) | 0.86 |
| HE | 4 (6.1) | 3 (4.2) | 1 (1.0) | 0.18 |
| SBP | 1 (1.5) | 0 (0) | 0 (0) | 0.27 |
| Varices | 6 (9.1) | 4 (5.6) | 7 (7.0) | 0.73 |
| Diabetes | 41 (62.1) | 38 (52.8) | 62 (62.0) | 0.41 |
| Number of tumors | 1.0 [1.0, 1.0] | 1.0 [1.0, 1.0] | 1.0 [1.0, 1.0] | 1.0 |
| Total tumor size (cm) | 1.5 [1.3, 1.7] | 1.5 [1.3, 1.7] | 1.5 [1.3, 1.8] | 0.18 |
| Largest tumor (cm) | 1.5 [1.3, 1.7] | 1.5 [1.3, 1.7] | 1.5 [1.3, 1.8] | 0.18 |
| Cirrhosis Comorbidity (CirCom)** | |  |  | 0.31 |
| 0 | 4 (6.1) | 2 (2.8) | 7 (7.0) |  |
| 1+0 | 19 (28.8) | 19 (26.4) | 17 (17.0) |  |
| 1+1 | 15 (22.7) | 14 (19.4) | 24 (24.0) |  |
| 3+0 | 0 (0) | 2 (2.8) | 4 (4.0) |  |
| 3+1 | 24 (36.4) | 34 (47.2) | 47 (47.0) |  |
| 5+0 | 2 (3.0) | 0 (0) | 0 (0) |  |
| 5+1 | 2 (3.0) | 1 (1.4) | 1 (1.0) |  |
| MILES | 7.4 [6.8, 7.7] | 7.5 [7.2, 7.9] | 7.5 [7.1, 7.8] | 0.23 |
| ALBI Grade |  |  |  | 0.161 |
| Grade 1 | 25 (37.9) | 28 (38.9) | 47 (47.0) |  |
| Grade 2 | 34 (51.5) | 39 (54.2) | 51 (51.0) |  |
| Grade 3 | 7 (10.6) | 5 (6.9) | 2 (2.0) |  |
| Transplant | 0 (0) | 2 (2.8) | 8 (8.0) |  |
| Hepatectomy | 0 (0) | 18 (25.0) | 7 (7.0) |  |
| Ablation | 0 (0) | 58 (80.6) | 90 (90.0) |  |
| HCC Oral/IV | 17 (25.8) | 0 (0) | 25 (25.0) |  |
| Radiation | 13 (19.7) | 0 (0) | 14 (14.0) |  |
| Embolization | 52 (78.8) | 0 (0) | 87 (87.0) |  |
| Time to treatment | 117.5 [41.3, 282.3] | 72.5 [44.8, 158.8] | 322.0 [126.8, 724.0] | <0.001 |
| Deaths within three years post-HCC therapy | 43 (65.2) | 28 (38.9) | 45 (45.0) | 0.005 |

*Median (IQR) for continuous variables or N (%) for categorical variables

** The CirCom score is a co-morbidity index developed by Jepsen, et al^25^
