# Supplementary material for: Differential survival benefit of curative versus non-curative intent treatment in a real-world cohort with early and intermediate-stage hepatocellular carcinoma
Source: Hepatol Commun. 2026 Jan 29;10(2):e0891. doi: 10.1097/HC9.0000000000000891 (PMC12858220; doi:10.1097/HC9.0000000000000891)
Supplement: Supplementary file 3 [file hc9-10-e0891-s003.docx]

Supplementary Table 3. Clinical and demographic characteristics of patients with HCC, BCLC stage A

| Variable* | Noncurative  (N=295) | | Curative  (N=140) | | Both  (N=277) | | P-value |
| --- | --- | --- | --- | --- | --- | --- | --- |
| Age (years) | 65.0 [62.0, 69.0] | | 66.0 [62.0, 69.0] | | 66.0 [62.0, 68.0] | | 0.81 |
| Male | 292 (99.0) | | 139 (99.3) | | 274 (98.9) | | 1.0 |
| Race |  | |  | |  | | 0.79 |
| White | 168 (56.9) | | 81 (57.9) | | 172 (62.1) | |  |
| Black | 73 (24.7) | | 35 (25.0) | | 63 (22.7) | |  |
| Hispanic | 20 (6.8) | | 9 (6.4) | | 21 (7.6) | |  |
| Asian or Pacific Islander | 8 (2.7) | | 4 (2.9) | | 3 (1.1) | |  |
| Other/Unknown | 26 (8.8) | | 11 (7.9) | | 18 (6.5) | |  |
| AFP (ng/mL) | 11.0 [5.2, 53.1] | | 5.2 [3.3, 11.0] | | 6.8 [3.9, 16.5] | | <0.001 |
| INR | 1.1 [1.0, 1.3] | | 1.1 [1.0, 1.2] | | 1.1 [1.0, 1.2] | | 0.045 |
| Sodium (mmol/L) | 139 [137, 141] | | 139 [137, 140] | | 138 [136, 140] | | 0.51 |
| Albumin (g/dL) | 3.7 [3.3, 4.0] | | 3.7 [3.3, 4.1] | | 3.8 [3.4, 4.1] | | 0.06 |
| Platelet (1000/µL) | 128 [87, 175] | | 148 [101, 207] | | 148 [92, 193] | | 0.005 |
| Total Bilirubin (mg/dL) | 0.9 [0.6, 1.3] | | 0.7 [0.5, 1.3] | | 0.8 [0.6, 1.2] | | 0.09 |
| eGFR (mL/min/1.73 m^2^) | 91.9 [72.4, 99.7] | | 84.3 [72.4, 97.7] | | 87.7 [67.5, 98.3] | | 0.35 |
| Etiology |  | |  | |  | | 0.87 |
| EtOH | 46 (15.6) | | 22 (15.7) | | 38 (13.7) | |  |
| EtOH+HCV | 107 (36.3) | | 45 (32.1) | | 102 (36.8) | |  |
| HCV | 95 (32.2) | | 48 (34.3) | | 86 (31.0) | |  |
| NAFLD-NASH | 35 (11.9) | | 22 (15.7) | | 42 (15.2) | |  |
| Other | 12 (4.1) | | 3 (2.1) | | 9 (3.2) | |  |
| Ascites | 16 (5.4) | | 6 (4.3) | | 7 (2.5) | | 0.21 |
| HE | 9 (3.1) | | 5 (3.6) | | 6 (2.2) | | 0.68 |
| SBP | 3 (1.0) | | 1 (0.7) | | 0 (0) | | 0.27 |
| Varices | 15 (5.1) | | 9 (6.4) | | 17 (6.1) | | 0.80 |
| Diabetes | 149 (50.5) | | 84 (60.0) | | 169 (61.0) | | 0.03 |
| Cirrhosis Comorbidity (CirCom)** | |  | |  | | 0.13 | |
| 0 | 13 (4.4) | | 9 (6.4) | | 10 (3.6) | |  |
| 1+0 | 72 (24.4) | | 33 (23.6) | | 44 (15.9) | |  |
| 1+1 | 69 (23.4) | | 38 (27.1) | | 61 (22.0) | |  |
| 3+0 | 9 (3.1) | | 6 (4.3) | | 13 (4.7) | |  |
| 3+1 | 123 (41.7) | | 53 (37.9) | | 140 (50.5) | |  |
| 5+0 | 2 (0.7) | | 0 (0) | | 4 (1.4) | |  |
| 5+1 | 7 (2.4) | | 1 (0.7) | | 5 (1.8) | |  |
| Number of tumors | 1.0 [1.0, 1.0] | | 1.0 [1.0, 1.0] | | 1.0 [1.0, 1.0] | | 0.03 |
| Total tumor size (cm) | 3.1 [2.5, 4.0] | | 2.7 [2.2, 3.4] | | 2.9 [2.4, 3.8] | | <0.001 |
| Largest tumor (cm) | 2.8 [2.3, 3.5] | | 2.5 [2.1, 3.0] | | 2.6 [2.2, 3.3] | | 0.005 |
| MILES | 6.9 [6.6, 7.3] | | 7.2 [6.8, 7.6] | | 7.2 [6.8, 7.5] | | <0.001 |
| ALBI Grade |  | |  | |  | | 0.14 |
| Grade 1 | 99 (33.6) | | 59 (42.1) | | 109 (39.4) | |  |
| Grade 2 | 176 (59.7) | | 70 (50.0) | | 157 (56.7) | |  |
| Grade 3 | 20 (6.8) | | 11 (7.9) | | 11 (4.0) | |  |
| Transplant | 0 (0%) | | 7 (5.0) | | 38 (13.7) | |  |
| Hepatectomy | 0 (0%) | | 53 (37.9) | | 30 (10.8) | |  |
| Ablation | 0 (0%) | | 89 (63.6) | | 234 (84.5) | |  |
| HCC Oral/IV | 86 (29.2) | | 0 (0) | | 70 (25.3) | |  |
| Radiation | 34 (11.5) | | 0 (0) | | 39 (14.1) | |  |
| Embolization | 251 (85.1) | | 0 (0) | | 249 (89.9) | |  |
| Time to treatment | 87.0 [46.0, 175.0] | | 64.5 [45.0, 108.0] | | 274.0 [101.0, 650.0] | | <0.001 |
| Deaths within three years post-HCC therapy | 188 (63.7) | | 44 (31.4) | | 116 (41.9) | | <0.001 |

*Median (IQR) for continuous variables or N (%) for categorical variables

** The CirCom score is a co-morbidity index developed by Jepsen, et al^25^
