# Supplementary material for: Differential survival benefit of curative versus non-curative intent treatment in a real-world cohort with early and intermediate-stage hepatocellular carcinoma
Source: Hepatol Commun. 2026 Jan 29;10(2):e0891. doi: 10.1097/HC9.0000000000000891 (PMC12858220; doi:10.1097/HC9.0000000000000891)
Supplement: Supplementary file 4 [file hc9-10-e0891-s004.docx]

Supplementary Table 4. Clinical and demographic characteristics of patients with HCC, BCLC stage B

| Variable | Noncurative  (N=174) | Curative  (N=15) | Both  (N=52) | | P-value |
| --- | --- | --- | --- | --- | --- |
| Age (years) | 66.0 [62.0, 69.0] | 68.0 [65.5, 69.5] | 66.0 [63.8, 69.3] | | 0.58 |
| Male | 173 (99.4) | 15 (100) | 52 (100) | | 1.0 |
| Race |  |  |  | | 0.35 |
| White | 90 (51.7) | 9 (60.0) | 31 (59.6) | |  |
| Black | 52 (29.9) | 2 (13.3) | 8 (15.4) | |  |
| Hispanic | 11 (6.3) | 2 (13.3) | 5 (9.6) | |  |
| Asian or Pacific Islander | 6 (3.4) | 0 (0) | 1 (1.9) | |  |
| Other/Unknown | 15 (8.6) | 2 (13.3) | 7 (13.5) | |  |
| AFP (ng/mL) | 21.3 [6.5, 133.4] | 9.9 [6.6, 31.2] | 9.4 [4.0, 99.9] | | 0.08 |
| INR | 1.1 [1.0, 1.2] | 1.1 [1.0, 1.1] | 1.0 [1.0, 1.2] | | 0.007 |
| Sodium (mmol/L) | 138 [136, 140] | 137 [136, 139] | 138 [137, 140] | | 0.40 |
| Albumin (g/dL) | 3.6 [3.2, 3.9] | 3.7 [3.3, 4.2] | 3.7 [3.3, 4.1] | | 0.20 |
| Platelet (1000/µL) | 159 [107, 227] | 226 [181, 249] | 153 [109, 212] | | 0.05 |
| Total Bilirubin (mg/dL) | 0.8 [0.5, 1.3] | 0.7 [0.5, 1.3] | 0.7 [0.6, 1.0] | | 0.41 |
| eGFR (mL/min/1.73 m^2^) | 89.5 [67.2, 97.8] | 91.5 [75.5, 98.2] | 92.1 [71.5, 97.6] | | 0.92 |
| Etiology |  |  |  | | 0.42 |
| EtOH | 20 (11.5) | 1 (6.7) | 8 (15.4) | |  |
| EtOH+HCV | 67 (38.5) | 3 (20.0) | 14 (26.9) | |  |
| HCV | 61 (35.1) | 10 (66.7) | 20 (38.5) | |  |
| NAFLD-NASH | 21 (12.1) | 1 (6.7) | 9 (17.3) | |  |
| Other | 5 (2.9) | 0 (0) | 1 (1.9) | |  |
| Ascites | 9 (5.2) | 1 (6.7) | 1 (1.9) | | 0.44 |
| HE | 2 (1.1) | 0 (0) | 0 (0) | | 1.0 |
| SBP | 1 (0.6) | 0 (0) | 0 (0) | | 1.0 |
| Varices | 5 (2.9) | 1 (6.7) | 2 (3.8) | | 0.42 |
| Diabetes | 82 (47.1) | 8 (53.3) | 30 (57.7) | | 0.39 |
| Cirrhosis Comorbidity (CirCom) | |  |  | 0.16 | |
| 0 | 14 (8.0) | 1 (6.7) | 2 (3.8) | |  |
| 1+0 | 40 (23.0) | 3 (20.0) | 7 (13.5) | |  |
| 1+1 | 39 (22.4) | 2 (13.3) | 18 (34.6) | |  |
| 3+0 | 8 (4.6) | 0 (0) | 1 (1.9) | |  |
| 3+1 | 56 (32.2) | 9 (60.0) | 24 (46.2) | |  |
| 5+0 | 7 (4.0) | 0 (0) | 0 (0) | |  |
| 5+1 | 10 (5.7) | 0 (0) | 0 (0) | |  |
| Number of tumors | 2.0 [1.0, 3.0] | 1.0 [1.0, 2.0] | 1.0 [1.0, 2.3] | | 0.04 |
| Total tumor size (cm) | 8.1 [6.0, 10.1] | 6.5 [6.0, 6.9] | 6.9 [5.7, 8.5] | | 0.02 |
| Largest tumor (cm) | 5.6 [4.4, 8.0] | 6.2 [5.5, 6.6] | 5.4 [3.8, 7.0] | | 0.39 |
| MILES | 6.4 [5.9, 6.8] | 6.6 [6.4, 7.1] | 6.7 [6.2, 7.1] | | 0.009 |
| ALBI Score | -2.3 [-2.6, -1.8] | -2.7 [-2.9, -2.0] | -2.5 [-2.9, -1.9] | | 0.08 |
| ALBI Grade |  |  |  | | 0.02 |
| Grade 1 | 49 (28.2) | 8 (53.3) | 22 (42.3) | |  |
| Grade 2 | 111 (63.8) | 4 (26.7) | 26 (50.0) | |  |
| Grade 3 | 14 (8.0) | 3 (20.0) | 4 (7.7) | |  |
| Transplant | 0 (0%) | 0 (0) | 4 (7.7) | |  |
| Hepatectomy | 0 (0%) | 12 (80.0) | 18 (34.6) | |  |
| Ablation | 0 (0%) | 3 (20.0) | 35 (67.3) | |  |
| HCC Oral/IV | 92 (52.9) | 0 (0) | 16 (30.8) | |  |
| Radiation | 20 (11.5) | 0 (0) | 5 (9.6) | |  |
| Embolization | 134 (77.0) | 0 (0) | 48 (92.3) | |  |
| Time to treatment | 56.0 [34.0, 121.3] | 68.0 [27.0, 101.0] | 254.5 [118.3, 488.5] | | <0.001 |
| Deaths within three years post-HCC therapy | 131 (75.3) | 11 (73.3) | 30 (57.7) | | 0.05 |

*Median (IQR) for continuous variables or N (%) for categorical variables

** The CirCom score is a co-morbidity index developed by Jepsen, et al^25^
