# Supplementary material for: Differential survival benefit of curative versus non-curative intent treatment in a real-world cohort with early and intermediate-stage hepatocellular carcinoma
Source: Hepatol Commun. 2026 Jan 29;10(2):e0891. doi: 10.1097/HC9.0000000000000891 (PMC12858220; doi:10.1097/HC9.0000000000000891)

**Supplementary Figure 1.** Covariate balance across HCC treatment groups before and after inverse probability of treatment weighting (IPTW).


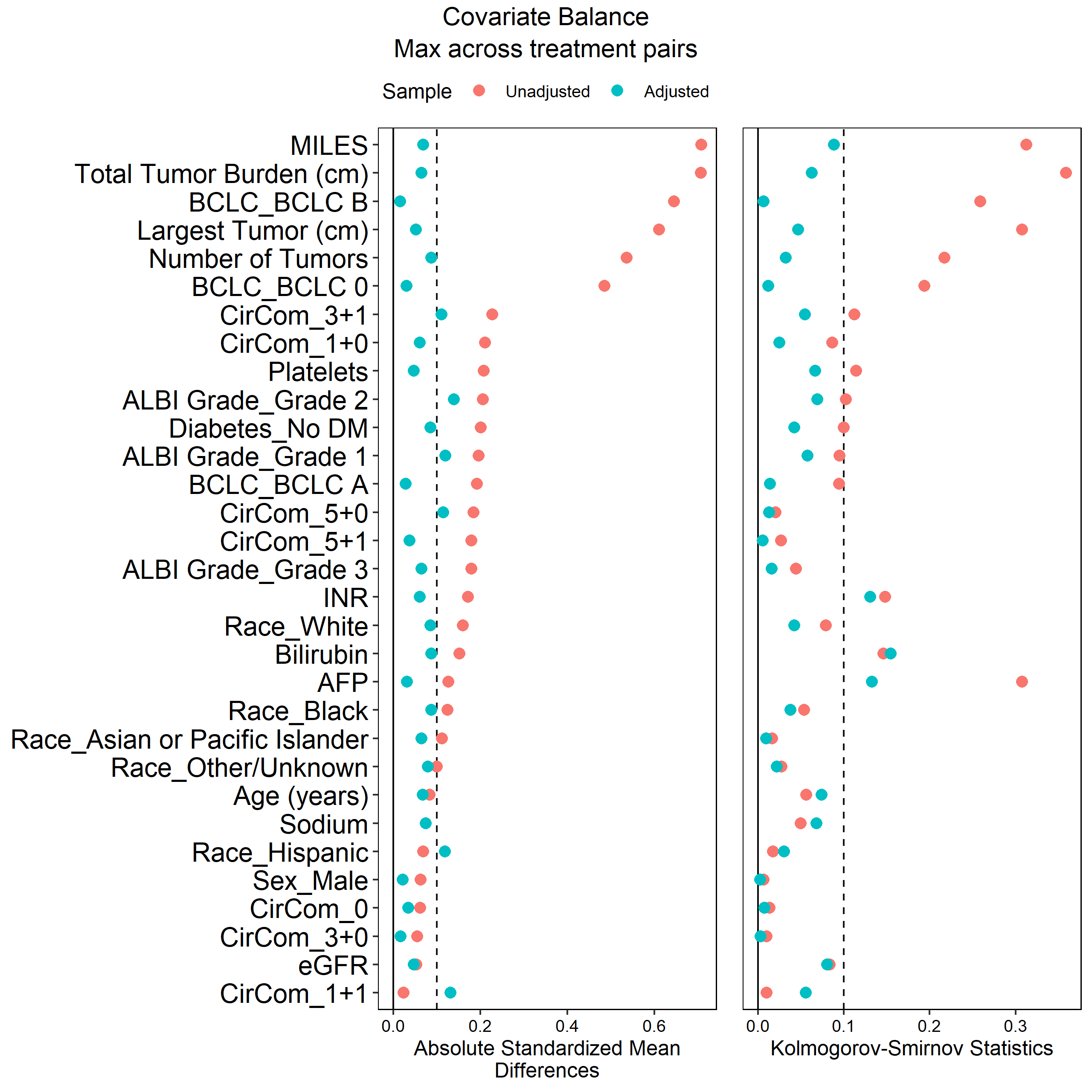

Supplement: Supplementary file 5 [file hc9-10-e0891-s005.docx]
