# Supplementary material for: Differential survival benefit of curative versus non-curative intent treatment in a real-world cohort with early and intermediate-stage hepatocellular carcinoma
Source: Hepatol Commun. 2026 Jan 29;10(2):e0891. doi: 10.1097/HC9.0000000000000891 (PMC12858220; doi:10.1097/HC9.0000000000000891)
Supplement: Supplementary file 6 [file hc9-10-e0891-s006.docx]

Supplementary Table 5. Hazard ratios for unadjusted and adjusted model

| **Model** | **BCLC** | **HCC Treatment** | **6 months** | **1 year** | **2 years** | **3 years** |
| --- | --- | --- | --- | --- | --- | --- |
|  | 0 | Noncurative | - | - | - | - |
| Unadjusted | 0 | Curative | 0.56 (0.34, 0.96) | 0.61 (0.40, 0.96) | 0.69 (0.50, 0.97) | 0.75 (0.59, 0.98) |
|  | 0 | Both | 0.44 (0.24, 0.76) | 0.48 (0.29, 0.80) | 0.57 (0.38, 0.84) | 0.64 (0.47, 0.87) |
|  | A | Noncurative | - | - | - | - |
|  | A | Curative | 0.50 (0.38, 0.65) | 0.55 (0.44, 0.69) | 0.63 (0.53, 0.75) | 0.70 (0.60, 0.80) |
|  | A | Both | 0.30 (0.21, 0.44) | 0.34 (0.25, 0.49) | 0.43 (0.32, 0.57) | 0.50 (0.39, 0.63) |
|  | B | Noncurative | - | - | - | - |
|  | B | Curative | 0.43 (0.26, 0.67) | 0.51 (0.34, 0.73) | 0.63 (0.46, 0.82) | 0.71 (0.55, 0.87) |
|  | B | Both | 1.23 (0.58, 2.18) | 1.16 (0.66, 1.67) | 1.09 (0.76, 1.34) | 1.06 (0.82, 1.22) |
|  | 0 | Noncurative | - | - | - | - |
| Adjusted  +  IPTW | 0 | Curative | 0.53 (0.28, 0.97) | 0.59 (0.34, 0.98) | 0.69 (0.46, 0.98) | 0.77 (0.55, 0.99) |
|  | 0 | Both | 0.70 (0.43, 1.11) | 0.75 (0.50, 1.09) | 0.82 (0.62, 1.05) | 0.87 (0.71, 1.04) |
|  | A | Noncurative | - | - | - | - |
|  | A | Curative | 0.41 (0.28, 0.60) | 0.45 (0.32, 0.64) | 0.54 (0.40, 0.71) | 0.61 (0.48, 0.76) |
|  | A | Both | 0.58 (0.43, 0.77) | 0.62 (0.48, 0.79) | 0.70 (0.58, 0.84) | 0.76 (0.65, 0.88) |
|  | B | Noncurative | - | - | - | - |
|  | B | Curative | 2.26 (1.49, 3.54) | 1.82 (1.34, 2.56) | 1.45 (1.20, 1.82) | 1.30 (1.13, 1.54) |
|  | B | Both | 0.44 (0.28, 0.70) | 0.49 (0.33, 0.73) | 0.58 (0.43, 0.79) | 0.65 (0.51, 0.84) |
