# Supplementary material for: Differential survival benefit of curative versus non-curative intent treatment in a real-world cohort with early and intermediate-stage hepatocellular carcinoma
Source: Hepatol Commun. 2026 Jan 29;10(2):e0891. doi: 10.1097/HC9.0000000000000891 (PMC12858220; doi:10.1097/HC9.0000000000000891)
Supplement: Supplementary file 7 [file hc9-10-e0891-s007.docx]

Supplementary Table 6: Hazard ratios for multivariable models with IPTW excluding patients who only received systemic chemotherapy

| **BCLC** | **HCC Treatment vs Noncurative** | **N** | **Deaths** | **6 months** | **1 year** | **2 years** | **3 years** |
| --- | --- | --- | --- | --- | --- | --- | --- |
| 0 | Noncurative | 60 | 37 | - | - | - | - |
| 0 | Curative | 72 | 28 | 0.59 (0.31, 1.17) | 0.64 (0.36, 1.13) | 0.72 (0.46, 1.08) | 0.79 (0.55, 1.05) |
| 0 | Both | 100 | 45 | 0.79 (0.49, 1.33) | 0.82 (0.55, 1.26) | 0.87 (0.65, 1.18) | 0.91 (0.74, 1.13) |
| A | Noncurative | 268 | 168 | - | - | - | - |
| A | Curative | 140 | 44 | 0.44 (0.31, 0.64) | 0.48 (0.35, 0.68) | 0.56 (0.43, 0.74) | 0.62 (0.50, 0.79) |
| A | Both | 277 | 116 | 0.62 (0.45, 0.85) | 0.66 (0.50, 0.87) | 0.72 (0.58, 0.90) | 0.78 (0.66, 0.92) |
| B | Noncurative | 140 | 98 | - | - | - | - |
| B | Curative | 15 | 11 | 2.59 (1.63, 4.26) | 2.05 (1.45, 2.98) | 1.59 (1.27, 2.04) | 1.39 (1.18, 1.68) |
| B | Both | 52 | 30 | 0.52 (0.31, 0.85) | 0.56 (0.36, 0.87) | 0.64 (0.45, 0.90) | 0.70 (0.53, 0.92) |
