# Supplementary material for: Differential survival benefit of curative versus non-curative intent treatment in a real-world cohort with early and intermediate-stage hepatocellular carcinoma
Source: Hepatol Commun. 2026 Jan 29;10(2):e0891. doi: 10.1097/HC9.0000000000000891 (PMC12858220; doi:10.1097/HC9.0000000000000891)
Supplement: Supplementary file 8 [file hc9-10-e0891-s008.docx]

Supplementary Table 7: Hazard ratios for multivariable models with IPTW excluding patients who received curative treatment followed by noncurative

| **BCLC** | **HCC Treatment**  **vs Noncurative** | **N** | **Deaths** | **6 months** | **1 year** | **2 years** | **3 years** |
| --- | --- | --- | --- | --- | --- | --- | --- |
| 0 | Noncurative | 66 | 43 | - | - | - | - |
| 0 | Curative TX | 72 | 28 | 0.55 (0.30, 1.01) | 0.61 (0.36, 1.00) | 0.71 (0.47, 1.00) | 0.78 (0.57, 1.00) |
| 0 | Noncurative-Curative | 52 | 19 | 0.49 (0.27, 0.93) | 0.55 (0.33, 0.94) | 0.66 (0.44, 0.96) | 0.74 (0.54, 0.97) |
| A | Noncurative | 295 | 188 | - | - | - | - |
| A | Curative | 140 | 44 | 0.40 (0.27, 0.58) | 0.44 (0.31, 0.63) | 0.53 (0.4, 0.70) | 0.61 (0.48, 0.76) |
| A | Noncurative-Curative | 166 | 53 | 0.39 (0.26, 0.54) | 0.43 (0.31, 0.58) | 0.52 (0.39, 0.66) | 0.60 (0.47, 0.73) |
| B | Noncurative | 174 | 131 | - | - | - | - |
| B | Curative TX | 15 | 11 | 2.44 (1.62, 3.88) | 1.90 (1.42, 2.64) | 1.47 (1.23, 1.8) | 1.30 (1.15, 1.5) |
| B | Noncurative-Curative | 39 | 24 | 0.34 (0.19, 0.56) | 0.39 (0.23, 0.62) | 0.48 (0.32, 0.70) | 0.57 (0.4, 0.76) |
